# A variant of death-receptor 3 associated with rheumatoid arthritis interferes with apoptosis-induction of T cell

**DOI:** 10.1074/jbc.M117.798884

**Published:** 2017-11-27

**Authors:** Akira Hashiramoto, Yoshitake Konishi, Koichi Murayama, Hiroki Kawasaki, Kohsuke Yoshida, Ken Tsumiyama, Kimie Tanaka, Masaru Mizuhara, Toshio Shiotsuki, Hitomi Kitamura, Koichiro Komai, Tomoatsu Kimura, Hideo Yagita, Kazuko Shiozawa, Shunichi Shiozawa

**Affiliations:** From the ‡ Department of Biophysics, Kobe University Graduate School of Health Science, Kobe 654-0142,; the § Department of Medicine, Rheumatic Diseases Unit, Kyushu University Beppu Hospital, Beppu 874-0838,; the ¶ Department of Orthopedic Surgery, Faculty of Medicine, University of Toyama, 3190 Gofuku, 930-0194 Toyama,; the ‖ Department of Immunology, Juntendo University School of Medicine, Tokyo 113-8431, and; the ** Department of Rheumatology, Hyogo Prefectural Kakogawa Medical Center, Kakogawa 675–8555, Japan

**Keywords:** apoptosis, arthritis, caspase, death domain, lymphocyte

## Abstract

Rheumatoid arthritis (RA) is a chronic polyarthritis of unknown etiology. To unravel the molecular mechanisms in RA, we performed targeted DNA sequencing analysis of patients with RA. This analysis identified a variant of the *death receptor 3* (*DR3*) gene, a member of the family of apoptosis-inducing *Fas* genes, which contains four single-nucleotide polymorphisms (SNPs) and a 14-nucleotide deletion within exon 5 and intron 5. We found that the deletion causes the binding of splicing regulatory proteins to *DR3* pre-mRNA intron 5, resulting in a portion of intron 5 becoming part of the coding sequence, thereby generating a premature stop codon. We also found that this truncated DR3 protein product lacks the death domain and forms a heterotrimer complex with wildtype DR3 that dominant-negatively inhibits ligand-induced apoptosis in lymphocytes. Myelocytes from transgenic mice expressing the human *DR3* variant produced soluble truncated DR3, forming a complex with TNF-like ligand 1A (TL1A), which inhibited apoptosis induction. In summary, our results reveal that a *DR3* splice variant that interferes with ligand-induced T cell responses and apoptosis may contribute to RA pathogenesis.

## Introduction

The death receptor 3 (DR3),^2^ also named WSL-1, Apo3, TNF-receptor-related apoptosis-mediating protein, lymphocyte-associated receptor of death (LARD), TR3, or tumor-necrosis factor receptor superfamily number 25 (TNFRSF25) (1), is a member of the tumor necrosis factor receptor (TNFR) superfamily that includes Fas, TNFR1, DR4, DR5, and DR6 (2–5). It contains four extracellular cysteine-rich motifs and a characteristic intracellular death domain capable of mediating either cellular apoptosis via TNFR-associated death domain protein (TRADD) followed by association with Fas-associated death domain or NFκB activation via TRADD followed by interaction with TNFR-associated factor 2 (TRAF2) (6, 7). The DR3 is expressed primarily in lymphocytes, upon activation with immune complex, Toll-like receptor, or inflammatory cytokines such as TNF or IL1β (2–5, 8–14). The ligand for DR3 is the TNF-like ligand 1A (TL1A), also known as TNF ligand superfamily member 15 (TNFSF15) (15). Studies have shown that TL1A expression correlates with the presence and severity of mucosal tissue inflammation of patients with ulcerative colitis and Crohn's disease (9, 10, 16–22). Studies also suggested the importance of TL1A signaling in the pathogenesis of autoimmune or inflammatory diseases such as rheumatoid arthritis (8, 12, 13, 23–26), psoriasis (27, 28), primary biliary cirrhosis (29, 30), ankylosing spondylitis (31, 32), and allergic lung inflammation (33, 34), however, the disease mechanisms involving TL1A and its receptor DR3 especially in humans remain elusive.

In the present study, we investigated the role of DR3 by directly sequencing the entire *DR3* genome to identify a novel genetic variant of DR3 encoding a truncated DR3 that inhibits ligand-induced apoptosis in a dominant-negative fashion in patients with rheumatoid arthritis (RA). We studied the expression of the variant DR3, composition of death receptor trimer, and the contribution of variant DR3 to the induction of apoptosis and arthritis.

## Results

### Variant of DR3

**Figure 1.**
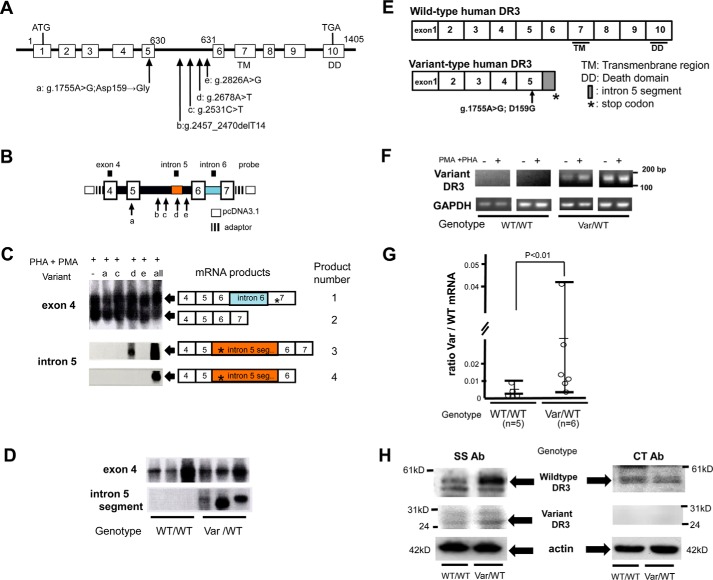
**Polymorphisms of DR3 in lymphocytes.** *A,* location of polymorphisms in relationship to transmembrane (*TM*) and death domains (*DD*) found in patients with RA (*n* = 50). *Numbered boxes* represent exons. The polymorphisms were: *a,* g.1775A>G (rs11800462); *b,* g.2457_2382delT14; *c,* g.2531C>T (rs3138153); *d,* g.2678A>T (rs3138155); and *e,* g.2826A>G (rs3138156) from the first nucleotide of ATG sequence. These polymorphisms corresponded to the previous nomenclature, numbered from the first base of exon 1 as follows: nt 564 (A → G); Asp-159 → Gly, nt 630 + 622 (del 14), nt 631–538 (C → T), nt 631–391 (A → T), and nt 631–243 (A → G), respectively. GenBank accession numbers for *DR3* cDNA, authentic genomic *DR3*, and variant genomic *DR3* are U746116, AB051850.1, and AB051851.1. *B,* construction of pcDNA3.1/DR3 minigene. *C,* Southern blotting of the lysate of Jurkat cell transfected with pcDNA3.1/DR3 minigene and stimulated with PMA and PHA. Termination codons are indicated by *asterisks. D,* RT-PCR analysis of lymphocytes from rheumatoid patients with (*Var*/*WT*) or without (*WT*/*WT*) variant *DR3* and stimulated with PMA and PHA. *E,* wildtype human DR3 and variant-type human DR3 mRNA. The exon 7 codes transmembrane region and exon 10 codes the death domain. *F,* RT-PCR analysis of variant *DR3* mRNA amplified from patients' lymphocytes using primers encompassing exons 4–5 and intron 5 (putative exon; g.2636∼g.2792). *G,* quantitative RT-PCR analysis of wild-type *DR3* (WT) (product 2 in *c*) (*n* = 5) and variant *DR3* (Var) (products 3 and 4 in *c*) (*n* = 6) expressed as Var/WT ratio in the patients. *H,* Western blot analysis of variant and wildtype DR3 in the lymphocytes from rheumatoid patients with (*Var/WT*) or without (*WT/WT*) variant *DR3. SS Ab* indicates an anti-DR3 (SS) antibody reactive against the extracellular N-terminal 25–46 amino acids. *CT Ab* indicates an anti-DR3 (*CT*) antibody reactive against the intracellular C-terminal 398–417 amino acids including the death domain.

We directly sequenced the entire DR3 genome of patients with RA, and found a variant of the *DR3* gene that contains four single nucleotide polymorphisms (SNPs) and one 14-nucleotide deletion, which we termed polymorphisms a, c, d, e, and b (Fig. 1*A*; SNPa, rs11800462; SNPb, rs763855745; SNPc, rs3138153; SNPd, rs3138155; SNPe, rs3138156, as registered GenBank^TM^ accession No. AB051850.1 to DR3 and AB051851.1 to variant DR3).

In nature, the majority of DR3-positive PBLs are T cells (Fig. S1), and the *DR3* gene splice variants as expressed in the lymphocytes of Caucasians typically skip between exons 4 and 5 (designated LARD4, -5, and -6) (4). Thus, we studied the expression of variant *DR3* by constructing a *DR3* minigene encompassing exons 4–7 (Fig. 1*B*), and transfected it into Jurkat cells to detect mRNA for 4 splice variants using reverse transcription-PCR (RT-PCR) followed by Southern blotting (Fig. 1*C*). Product 1 includes intron 6 in its coding sequence, leading to a premature stop codon (*asterisks*) emerging at exon 7 (designated LARD2) (5). According to Screaton *et al.* (5), this LARD2 protein was not detectable in Western blots of human lymphocytes. We also did not detect this using RT-PCR, and thus, this variant was considered sterile. Products 3 and 4 contained a portion of intron 5, g.2636–g.2792 in the coding sequence, leading to a premature stop codon (*asterisks*) and the generation of a truncated DR3 molecule. The inserted segment of intron 5 could potentially function as an exon and polymorphism d was responsible for this insertion (Fig. 1*C*). Indeed, RT-PCR analysis showed that this segment of intron 5 could be detected as a putative exon in lymphocytes from patients having variant *DR3* genotype after stimulation with phorbol myristate acetate (PMA, 20 ng/ml) and phytohemagglutinin (PHA, 1 μg/ml) for 48 h (Fig. 1*D*). The *DR3* variant was amplified from the lymphocytes of patients carrying the variant genotype. *DR3* mRNA variants such as LARD3–5 (5) were difficult to detect by Northern blotting and other methods (35), but can be detected by RT-PCR (5). RT-PCR primers encompassing exon 4 and intron 5 were used to detect the putative exon encoded by intron 5 that is induced upon stimulation (Fig. 1, *E* and *F*). As shown in Fig. 1*G*, quantitative PCR specific for the putative exon encoded by intron 5 showed significantly higher amounts of variant *DR3* mRNA (products 3 and 4 of Fig. 1*C*) in lymphocytes from patients with variant *DR3 as* compared with normal. In addition, cell lysates from patients' lymphocytes were subjected to Western blotting using anti-DR3 (SS) antibody and anti-DR3 (CT) antibody. Because SS antibody could recognize the extracellular portion of DR3, the truncated variant DR3 was detected as a 26-kDa protein in individuals with the variant *DR3* genotype (Fig. 1*H*).

### Splicing regulatory protein binding to the variant DR3 pre-mRNA

**Figure 2.**
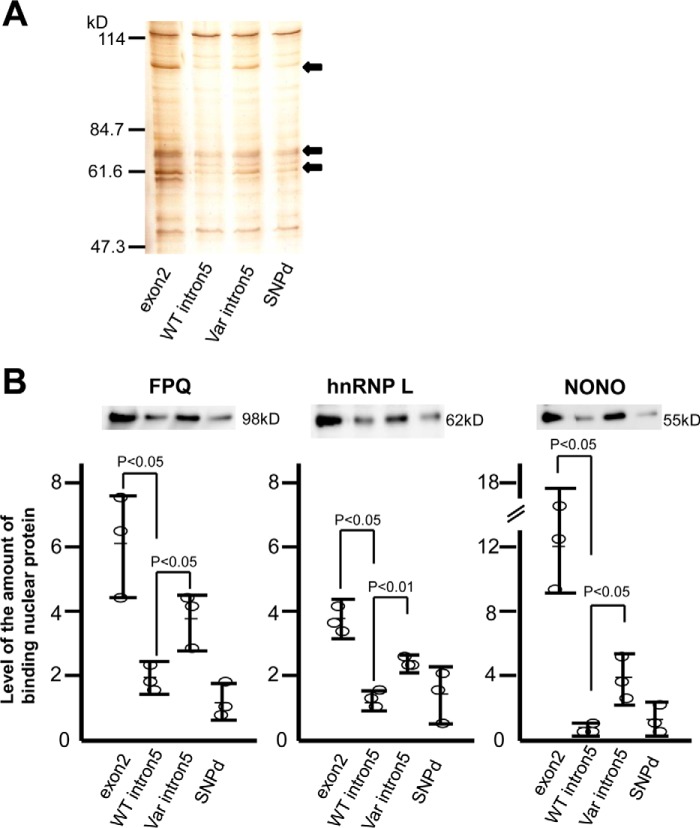
**Splicing regulatory proteins bind to the *DR3* intron 5.** *A,* silver staining of nuclear proteins bound to exon 2, WT intron 5, MT intron 5, and SNPd pre-mRNA. 100-, 70-, and 60-kDa proteins strongly bound to exon 2 and MT intron 5 as compared with WT intron 5 (*arrows*). *B,* Western blotting analysis of nuclear proteins bound to exon 2, WT intron 5, MT intron 5, and SNPd pre-mRNA using anti-SFPQ, hnRNP L, or NONO antibodies. The level of SFPQ, hnRNP L, and NONO bound to pre-mRNA were enhanced in exon 2 and MT intron 5 as compared with WT intron 5. Samples were assayed in three independent experiments and results were expressed as mean ± S.D.

The 5′-end of intron 5 (putative exon) contains the nucleotide sequence “tttagtaga,” where the first “tag” corresponds to a 3′ consensus splice sequence (GenBank accession AB051851.1). A consensus polypyrimidine tract also exists in the upstream of the tag sequence, suggesting that insertion of sequences from intron 5 into the variant DR3 mRNA could be due to the involvement of splicing modulators. Such modulators have been shown to alter gene expression in *Drosophila* by binding to sequences in a putative exon (36). Therefore, we analyzed nuclear proteins bound to *DR3* pre-mRNA intron 5. We first compared the amounts of nuclear proteins bound to variant intron 5 and wildtype intron 5 pre-mRNA by silver staining. We detected the proteins of 100, 70, and 60 kDa that bound more strongly to exon 2 pre-mRNA than to wildtype intron 5. These same proteins also bound more strongly to variant intron 5 pre-mRNA than to wildtype intron 5. The amounts of nuclear proteins bound to wildtype intron 5 and SNPd were comparable (Fig. 2*A*). We next analyzed the nuclear proteins bound to variant intron 5 pre-mRNA by using mass spectrometry. We identified 100-, 70-, and 60-kDa nuclear proteins as three splicing factors: proline- and glutamine-rich (SFPQ), the heterogeneous nuclear ribonucleoprotein L (hnRNP L), and the non-POU domain-containing octamer-binding protein (NONO), selectively bound to the variant intron 5 *versus* wildtype intron 5 (*p* < 0.05) encompassing SNPd (Fig. 2*B*). This would suggest a model in which mutation in the DR3 sequence lead to abnormal binding of splicing regulatory proteins to *DR3* intron 5, thereby inducing pre-mRNA expression and insertion of a portion of intron 5 into the resulting mRNA product.

### Molecular assembly of DR3

**Figure 3.**
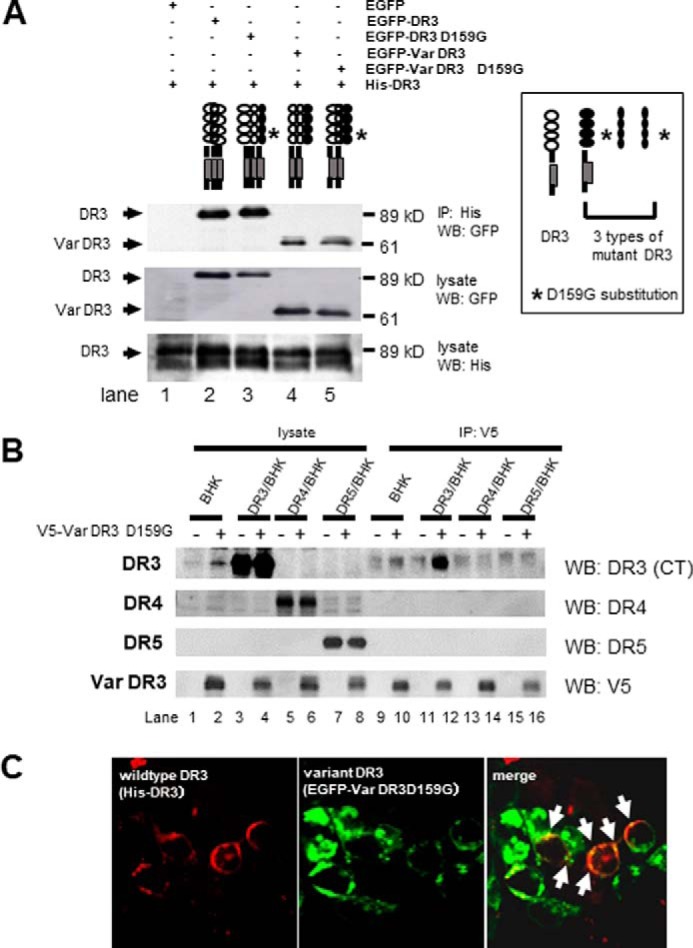
**Assembly of variant and authentic DR3 molecules in a transient expression system.** *A,* assembly of variant and authentic DR3 molecules in a transient expression system. Lysates of 293T cells were immunoprecipitated (*IP*) and subjected to Western blotting (*WB*) with anti-His monoclonal antibody and anti-GFP antibody. His-tagged wildtype DR3 (*His-DR3*) could assemble with DR3 having an amino acid substitution due to polymorphism a (*DR3-D159G*), and with variant DR3 with (*Var DR3-D159G*) or without (*Var DR3*) this amino acid substitution. *B,* assembly of truncated V5-tagged variant DR3-D159G (*V5-VarDR3-D159G*) with DR3, DR4, or DR5 expressed in stable BHK transformants. Lysates were immunoprecipitated with anti-V5 monoclonal antibody and blotted with an anti-DR3 (*CT*) antibody specifically recognizing the death domain, anti-DR4 antibody, anti-DR5 antibody, or an anti-V5 monoclonal antibody recognizing V5-Var DR3-D159G. *C,* immunofluorescent microscopy showing assembly of variant DR3 (*EGFP-Var DR3-D159G*; *green*) with wildtype DR3 (*His-DR3*; *red*) on the cell surface of 293 T cells (*yellow*).

We next examined assembly of DR3 complexes containing variant and wildtype DR3 by expressing in 293T cells. Immunoblot analysis showed that His-tagged wildtype DR3 (His-DR3) co-immunoprecipitated with EGFP-DR3D159G (wildtype DR3 with a D159G substitution, *i.e.* polymorphism a) (Fig. 3*A, lane 3*). His-DR3 also co-immunoprecipitated with the variant DR3 gene product, regardless of whether it contained the D159G substitution (EGFP-Var DR3-D159G) or not (EGFP-VarΔDR3) (Fig. 3*A, lanes 4* and *5*). Experiments in stably transformed BHK cells indicated that the variant DR3 specifically co-immunoprecipitated with wildtype DR3 (Fig. 3*B*). Immunofluorescent microscopy showed that truncated EGFP-Var DR3-D159G was co-expressed with wildtype His-DR3 on the cell surface (Fig. 3*C*). Thus, based on the mutations described above, truncated DR3 molecule lacking both death domain and transmembrane portion did assemble with the wildtype DR3 molecule to make a heterozygous trimmer complex.

### Defect in apoptosis induction

**Figure 4.**
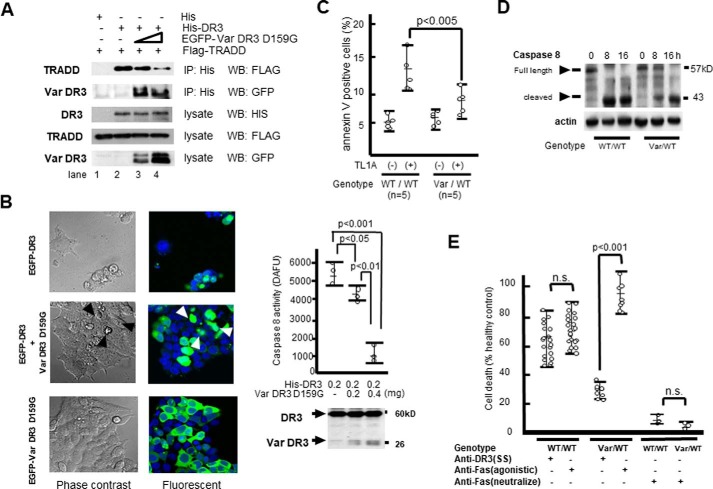
**Studies of apoptosis-induction.** *A,* assembly of authentic His-DR3 with FLAG-tagged TRADD (*Flag-TRADD*) in 293T cell was inhibited by the addition of truncated variant EGFP-Var DR3-D159G (0.1 and 0.2 μg in *lanes 3* and *4*, respectively) as examined by immunoprecipitation (*IP*) and Western blotting (*WB*). *B,* microscopic visualization of apoptosis. Apoptosis of 293T cells expressing wildtype DR3 (0.5 μg) (*left top*), cells co-expressing wildtype and variant DR3-D159G (*left middle*, Var DR3-D159G: 0.5 μg), and cells expressing Var DR3-D159G (*left bottom*). Variant DR3-D159G showed the resistance for apoptosis induced by wildtype DR3. *Arrowheads* indicated a small number of apoptotic cells existed in case with co-transfection of wildtype and variant DR3. Cells were stained with anti-GFP antibody and observed by confocal laser scanning microscopy. *Right,* caspase 8 activities in His-DR3 and/or Var DR3-D159G-transfected 293T cells (*n* = 3). Caspase 8 activity and the amount of expressed DR3 protein were simultaneously measured by Western blotting using anti-DR3 (SS) antibody 24 h after transfection. Arbitrary fluorescence units (Δ*AFU*) were calculated as: (caspase activity of DR3 vector) − (caspase activity of empty vector. Samples were assayed in three independent experiments. *C,* lymphocytes of patients with (Var/WT) or without (WT/WT) the variant *DR3* genotype were stained for Annexin V-positive apoptotic cells after stimulation with TL1A (200 ng/ml) for 16 h and observed by flow cytometry. *D,* inhibition of ligand-induced caspase 8 cleavage. Lymphocytes from rheumatoid patients with (Var/WT) or without (WT/WT) variant *DR3* genotype were stimulated with TL1A (200 ng/ml) for 8–16 h. *E,* lymphocytes were stimulated with anti-DR3 (SS) antibody, anti-Fas monoclonal antibody (CH-11; agonistic antibody) or anti-Fas polyclonal antibody (HPA027444; neutralize antibody) for 48 h. Samples were collected from patients with WT/WT genotype (*n* = 20) and Var/WT genotype (*n* = 8). Among them, 3 patients with each genotype were tested by anti-Fas polyclonal antibody as controls. Cell death was analyzed by flow cytometry and quantitated as (1 − (viable cells recovered from wells with test antibody)/(viable cells recovered from wells with control antibody or buffer)] × 100%.

With reference to prior discovery on the contribution of apoptosis defects to the pathogenesis of autoimmunity or arthritis (36–43), we studied the effect of the variant DR3 on death signaling (44). We transfected 293T cells with the vectors expressing wildtype DR3 and TRADD in combination with increasing amounts of variant DR3 (EGFP-Var DR3-D159G). We observed that His-DR3 co-immunoprecipitated with FLAG-tagged TRADD (FLAG-TRADD) (Fig. 4*A, lane 2*). The amount of TRADD co-precipitating with His-DR3 gradually decreased with increasing amounts of EGFP-Var DR3-D159G added (Fig. 4*A, lanes 3* and *4*), indicating that the variant DR3 dominantly interfered with the assembly of wildtype DR3 and TRADD, thereby inhibiting apoptotic signal transduction. This is consistent with the finding that truncated DR3, which lacks the normal death domain, does not bind TRADD (3). To verify the effect of Var DR3-D159G on apoptosis induction, we transfected 293T cells with wildtype DR3 and Var DR3-D159G, tagged with GFP. Cells transfected with wildtype DR3 displayed a morphological alteration typical of cells undergoing apoptosis (Fig. 4*B, top*). However, in the case of transfection with Var DR3-D159G, cells did not display any apoptotic changes (Fig. 4*B, bottom*), whereas a small number of apoptotic cells existed in the case of co-transfection with wildtype and Var DR3-D159G (Fig. 4*B, middle*, *arrowheads*). Caspase-8 activity was decreased as increasing amounts of variant DR3-D159G were transfected (Fig. 4*B*, *right*). We also observed that ligand-induced apoptosis by TL1A (15) was defective in the lymphocytes of patients having the variant DR3 genotype, as shown by annexin V-staining (Fig. 4*C*) and Western blotting for time-dependent caspase 8 cleavage (Fig. 4*D*). Furthermore, apoptosis induction in lymphocytes by an agonistic anti-DR3 (SS) antibody was defective in patients harboring variant *DR3* (*n* = 8) as compared with healthy controls (*n* = 20) (Fig. 4*E*).

### Studies of transgenic mice expressing variant type human DR3 gene

**Figure 5.**
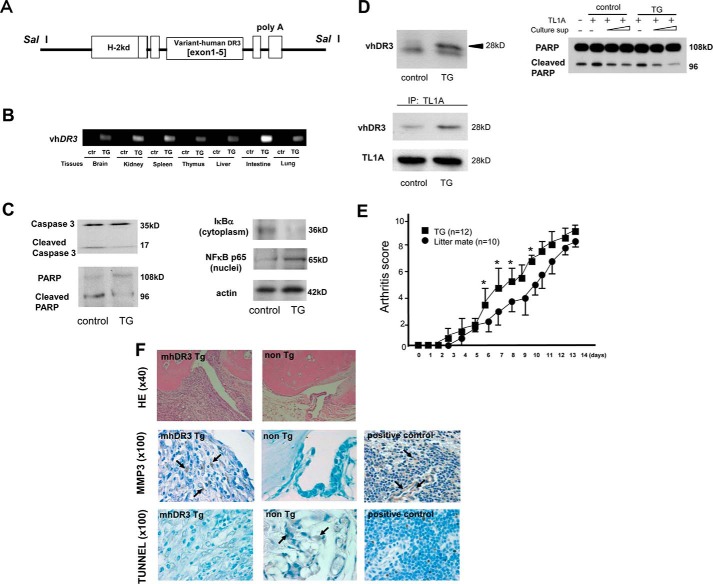
**Studies of TG mice expressing variant-type human *DR3* gene.** *A,* the construction of genes injected into C57BL/6 mice. *B,* RT-PCR of variant human *DR3* mRNA (vh*DR3*) in organs from TG or littermate (*ctr*) mice. *C,* Western blot of Caspase 3, PARP, IκBα, and NFκB p65 after TL1A (300 ng/ml) stimulation overnight followed by cycloheximide (10 μg/ml) treatment for 1 h. *D,* functional add-back assay of vhDR3. *Left upper*, detection of vhDR3 protein. A truncated DR3 molecule was specifically detected in vh*DR3*-TG myelocytes. *Lower left*, interaction between soluble vhDR3 and TL1A. TL1A-bounded soluble vhDR3 molecule from culture supernatants of TG myelocytes were analyzed by Western blotting after immunoprecipitation using anti-TL1A Abs. *Right*, inhibition of PARP cleavage by soluble vhDR3. TF-1 cells were stimulated overnight with TL1A (300 ng/ml) and culture supernatant containing soluble vhDR3 from TG or littermate myelocytes. *E,* evaluation of collagen-induced arthritis. Assessment of arthritis was made until 14 days after the 2nd immunization (score range: 0–12, *, *p* < 0.01). *F,* histological evaluation of collagen-induced arthritis joints including HE staining, immunohistochemistry for MMP3, and TUNNEL staining (day 14). Positive control for MMP3 and TUNNEL were mouse spleen and rat thymus, respectively.

We generated the transgenic (TG) mice expressing a variant-type human *DR3* gene (vhDR3 mice) (Fig. 5, *A* and *B*). Splenocytes from TG or littermate mice were treated with cycloheximide (10 μg) for 1 h, followed by treatment with 300 ng/ml of human recombinant TL1A overnight. Because cleavage of Caspase 3 and poly(ADP-ribose) polymerase (PARP) is considered hallmarks of apoptosis (44), they were determined by Western blots. As shown in Fig. 5*C* (*left*), splenocytes from TG mice did not show cleavages of both Caspase 3 and PARP, whereas cleaved substrates were clearly detected in littermate' splenocytes. Furthermore, because DR3 is also able to induce NFκB activation via TRADD, we studied TL1A-induced NFκB activation to find that although IκB degradation and nuclear translocation of NFκB p65 were operated minutely in wildtype mice, those were disturbed in TG splenocyte (Fig. 5*C, right*).

Next, myelocytes from vh*DR3*-TG mice and littermate control mice were cultured *in vitro*, and subjected to immunoblotting with anti-human DR3 antibodies. The vhDR3 molecule was specifically detected as a truncated protein of 28 kDa (Fig. 5*D, left upper*). When supernatants were subjected to immunoprecipitation with anti-TL1A Abs, soluble vhDR3 could be detected in the vhDR3-TG supernatants, indicating the formation of a complex between vhDR3 and human TL1A (Fig. 5*D, left lower*). To further examine the effect of vhDR3 on TL1A-induced signaling for cell death, TF-1 cells were stimulated overnight with 300 ng/ml of TL1A in the presence of culture supernatants from TG or littermates myelocytes. As sown in Fig. 5*D* (*right*), the culture supernatants from TG mice did block the TL1A-induced cleavage of PARP dose-dependently.

Finally, type II collagen-induced experimental arthritis was induced in vh*DR3-*TG mice (*n* = 12) and littermates (*n* = 10), and the arthritis score of vh*DR3*-TG mice was significantly higher than those of littermates on days 6–10 (*p* < 0.01) (Fig. 5*D*). We found strong inflammatory changes, including layered thickening of synovial cells and massive invasion of lymphocytes, and up-regulated expression of MMP3 in the joint of TG mice, whereas TUNNEL positive cells were not detected, indicating that apoptosis-induction was disturbed by vhDR3 (Fig. 5*E*).

### Variant DR3 gene in rheumatoid arthritis patients

The variant *DR3* gene as examined by the presence of the polymorphism d was found in 15 of 611 (2.45%) anti-citrullinated protein antibody (ACPA)-positive patients with RA, 15 of 538 (2.79%) ACPA- and rheumatoid factor-positive patients with RA, and 1 of 138 (0.72%) ACPA-negative patients with RA. The *DR3* variant gene was found in 2 of 547 (0.37%) healthy control individuals. The odd's ratio and relative risk as compared with healthy controls were 6.86 and 6.71 for ACPA-positive patients with RA, and those were 7.82 and 7.63 for ACPA- and rheumatoid factor-positive patients with RA. When the patients who underwent joint replacement surgery were retrospectively compared, the frequency of variant *DR3* was 8 of 42 (19.0%) and 0 of 59 (0%) in patients with RA and osteoarthritis, respectively (*p* = 0.00058), suggesting that variant *DR3* may predispose to progressive joint destruction.

## Discussion

We identified a *DR3* polymorphism that is over-represented in patients with RA. The *DR3* variant, containing four SNPs and one 14-nucleotide deletion within exon 5 and intron 5, resulted in the insertion of a portion of intron 5 into the coding sequence, thereby generating a premature stop codon. We showed that several splicing factors SFPQ, hnRNP L, and NONO selectively bound to the mutated DR3 intron 5 pre-mRNA *versus* wildtype intron 5. SFPQ is a factor required early in spliceosome formation (45, 46), and the SFPQ–NONO splicing complex is involved in the binding of U4/U5/U6 tri-small nuclear ribonucleoprotein (47). The hnRNP L regulates the splicing pathway by binding to CA-rich motifs and activation-responsive sequence motifs (48, 49). Because genetic mutations near the splice sites can alter splicing patterns (50, 51) and splicing enhancers in introns can promote exon insertion (52), we envisioned that these splicing regulatory proteins bound to the mutated DR3 intron 5 pre-mRNA might promote the insertion of a portion of intron 5 into the final mRNA to generate the variant DR3. A very similar splice variant of the *Fas* gene that contains a portion of intron 5 as a putative exon has been reported in cutaneous T cell lymphoma (53).

Previous studies have shown that the death receptor containing DR3 efficiently transmits the death signal via complex formation with TRADD and Fas-associated death domain (3, 54). Borysenko *et al.* (56) have pointed out in patients with RA that one polymorphism in the *DR3* variant, that we named “polymorphism a,” *i.e.* g.1755A→G; Asp-159 → Gly (GenBank accession numbers AB051851.1 and AB308321.1), occurs in a region critical for the structural integrity of ligand–receptor complexes (55). This could lead to destabilization of the variant DR3. We now show that a DR3 variant devoid of its own death domain can assemble with authentic DR3 to inhibit the apoptosis induction in the lymphocytes of rheumatoid patients. This is consistent with a previous finding showing that a truncated DR3 lacking the death domain did not bind TRADD (3). We in fact found that the lymphocytes of patients expressing the variant DR3 were highly resistant to ligand-induced apoptosis (Fig. 4*E*). Furthermore, in experiments, the myelocytes of mice overexpressing the human truncated DR3 molecule produced a soluble protein (vhDR3) that inhibited apoptosis induction *in vitro* in a similar fashion to Fas (57, 58), and experimental collagen-induced arthritis was enhanced *in vivo* in transgenic mice expressing vh*DR3*. Thus, whereas DR3 is characterized by the existence of plenty of splice variants (1, 3, 5), a novel mechanism of *DR3* splice variants that interferes with ligand-induced apoptosis seems to operate in the disease process in RA. Furthermore, the following findings may add evidence to the contribution of genetic or functional defects of DR3 to the pathogenesis of RA. *DR3* gene duplication was over-represented in RA (59). The CpG islands in the promoter region of the *DR3* gene were hypermethylated, where inverse correlation existed in the levels of serum DR3 protein and promoter CpG methylation in rheumatoid synovia (60). In relationship to DR3, TRAIL-induced apoptosis has been shown to be defective in the monocytes of RA patients (60), and the decoy receptor 3 that protects cells from Fas-induced apoptosis was also over-represented in RA (61, 62).

## Experimental procedures

### Patients and samples

Peripheral blood samples from patients with RA and healthy controls were obtained from the Konan-Kakogawa hospital, Kobe University Hospital, with written informed consents, based on the approval by the institutional review board of Kobe University.

### DNA sequencing

Genomic DNA, obtained from PBL of patients with RA and healthy controls, was directly sequenced or sequenced after cloning into pT7Blue vector (Promega, Madison, WI).

### Minigene experiment

A portion of the *DR3* genome encompassing exons 4–7 was amplified, subcloned into the pcDNA3.1 vector (Promega), and transfected into Jurkat cells (Riken, Tokyo, Japan). Total RNA was extracted from cells stimulated with PMA (20 ng/ml) and PHA (1 μg/ml) for 48 h, amplified, and analyzed by RT-PCR and Southern blotting. cDNA was transferred to Hybon-N+ nylon membrane (Amersham Biosciences), and hybridized with the exon 4 probe 5′-GCCAGGCTGGTTTGTGGA-3′ or the intron 5 probe 5′-CTCATGCCTGTAATCCCAGC-3′. For site-directed mutagenesis, genomic DNA was amplified by AmpliTaq DNA polymerase using the primers 5′-GGGGTACCATCCGCTTCCTGCCCCAGCCAGGCTGGTTTGTGGAGTGC-3′ (forward) and 5′-CCGCTCGAGGGGCCACCTCCAGTGCCAGTGGCGGTATGTGTAGGTCAGG-3 (reverse), and the products were subcloned into pcDNA3.1 (Promega).

Site-directed mutagenesis to mutations a, c, d, and e was performed in a 25-μl reaction containing 40 μm of each dNTP, reaction buffer, 1.25 units of *Pfu* Turbo DNA polymerase (Stratagene), 62.5 ng each of the sense and antisense primers, and 50 ng of the wildtype vector. Primers were: 5′-GGTTCCCGCAGAGGTACTGACTGTGGGA-3′ (sense) and 5′-TCCCACAGTCAGTACCTCTGCGGGAACC-3′(antisense).

Mutation “a” was introduced at underlined bases. Other mutations, c, d, and e, were introduced likewise.

### Genotyping

Because four SNPs and one 14-nucleotide deletion were always haplotypic and maintained a linkage disequilibrium, we amplified human DR3 SNPd for genotyping and confirmed all of the PCR products by sequencing. For the 1st PCR of human DR3 SNPd, genomic DNA was extracted from human PBL and amplified by the primer set: forward, 5′-TCAAGTGATTCTCCTGCCTC-3′(2550–2569: accession number AB051850.1), and reverse, 5′-AGGACAGGGATTTTGCCTAT-3′ (2328–2847: accession number AB051850.1). Using the 1st PCR product as a template, a 2nd PCR was performed with the primer set: forward, 5′-ACTACAGGAGCCCACCACCA-3′ (2589–2608: accession number AB051850.1); and reverse, 5′-TCCCCTGTTGCATCCCCA-3′ (2806–2823: accession number AB051850.1).

### Statistical analysis

Data are expressed as the mean ± S.D., and Student's *t* test and Fisher's exact test were used for statistical analyses. All statistical tests were two-sided and were performed at an α level of 0.05.

### Study of splicing regulatory proteins

Wildtype (wt) intron 5, variant type (var) intron 5 having mutations of SNP b, c, d, and e (Var intron 5) and wt exon 2 in the human *DR3* gene were amplified from PBL of a patient with RA by RT-PCR. Amplified products used as templates to generate RNA using ScriptMAX^TM^ Thermo T7 Transcription Kit (TOYOBO CO., Osaka, Japan). RNA-binding nuclear proteins were isolated using the synthesized RNA (400 pmol) and the 3′-biotinylated single strand (ss) oligonucleotide (500 pmol, Sigma) using the MACS^TM^ Streptavidine Kit (Miltenyi Biotec, Bergisch Gladbach, Germany). RNA-binding nuclear proteins were resolved by SDS-PAGE, visualized using the Silver StainII Kit from Wako (Wako Pure Chemical Industries, Osaka, Japan).

Antibodies used for Western blotting were: mouse anti-SFPQ monoclonal Ab, mouse anti-hnRNP L monoclonal Ab, and rabbit anti-NONO polyclonal Ab were purchased from Novus Biologicals (Littleton, CO), Santa Cruz Biotechnology (Santa Cruz, CA), and ProteinTech Group (Chicago, IL), respectively. The levels of SFPQ, hnRNP L, and NONO proteins bound to RNA were determined by semiquantification of digitally captured image using the public domain NIH Image program. Values were normalized to the level of nuclear protein bound to wt intron 5 pre-mRNA.

### mRNA analysis

For RT-PCR, total RNA was extracted from lymphocytes (1 × 10^6^ ml^−1^) after stimulation with PMA (20 ng/ml) and PHA (1 μg/ml) for 48 h. For quantitative PCR, total RNA was extracted from lymphocytes (1 × 10^6^ ml^−1^) after stimulation with PHA (1 μg/ml) and recombinant human interleukin-2 (rhIL-2, 20 ng ml^−1^) for 48 h and then with rhIL-2 (20 ng ml^−1^) for 11 days.

Probes used for RT-PCR were: 5′-CCGGTGACTTCCACAAGAAG-3′ (forward, exon 2) and 5′-GTTCATAGAAG CCAGGCAGG-3′ (reverse, exon 5). For authentic and variant *DR3* mRNA: the latter probe was 5′-TCAGCAGTTCACCCTTCT-3′ (forward, exon 4, for 1st PCR), 5′-ACTGCCAACCATGCCTAGAC-3′ (forward, exon 4 for 2nd PCR), and 5′-CATCTTGGCTAACACGGT-3′ (reverse, intron 5, for 1st and 2nd PCR). TaqMan probes and primers for quantitative RT-PCR were 5′-TCCTGCCCCACGAGCACCCT-3′ (exons 5∼6), 5′-TGTTCCCGCAGAGATACTGACTG-3′ (forward, exons 4–5) and 5′-ACCCAGAACATCTGCCTCCA-3′ (reverse, exons 6–7) encompassing exons 4–7 for wildtype *DR3*; 5′-TGCCTGCCTGGCTTCTATGAACATG-3′ (exon 5), 5′-TGTTCCCGCAGAGATACTGACTG-5′ (forward, exons 4–5) and 5′-AACACGGTGAAACCCCGTCT-3′ (reverse, intron 5) encompassing exon 4 to intron 5 for mutant *DR3*. Glyceraldehyde-3-phosphate dehydrogenase (*GAPDH*) was used as a control.

### Study of DR3 assembly

Western blots were probed with anti-DR3 (SS) or anti-DR3 (CT; Imgenex, San Diego, CA) antibody. Anti-DR3 (SS) antibody, raised by multiple antigen technology (Amersham Biosciences) against N-terminal 25–46 amino acids, specifically bound DR3 and did not without cross-react against DR4, DR5, DcR1, DcR2, or Fas in BHK and WR19L transformants.

Variant and wildtype *DR3* cDNA was subcloned into the pT7Blue vector (Promega) and integrated into the pcDNA3.1/His vector (Invitrogen) to synthesize His-tagged DR3 (His-DR3). Likewise, enhanced green fluorescent protein (EGFP)-tagged authentic DR3, EGFP-tagged variant DR3 with (Var DR3-D159G) or without (Var DR3) amino acid substitution, and V5–Var DR3-D159G were synthesized. We transfected 293T or BHK cells expressing DR3, DR4, or DR5 with these vectors using Lipofectamine Plus reagent (Invitrogen). Lysates were immunoprecipitated with anti-His (Invitrogen) or anti-V5 (Invitrogen) monoclonal antibody, and reacted with antibodies to GFP (Santa Cruz Biotechnology), His, FLAG (Sigma), V5, DR4 (Santa Cruz), DR5 (Sigma), and DR3 (SS). Morphological changes and manner of molecular assembly of cells were detected by phase-contrast and fluorescent microscopy.

In Fig. 3*C*, wildtype DR3 (His DR3) was stained with tetramethylrhodamine isomer R (TRITC)-conjugated anti-rabbit antibody, and variant DR3 (EGFP-Var DR3-D159G) was stained with anti-GFP antibody. In Fig. 4*B*, both wildtype DR3 and variant DR3 were tagged by EGFP and stained with anti-GFP antibody, as referred to phase-contrast images.

### Induction of apoptosis

The *TRADD* cDNA was amplified using primers 5′-CGAGGCGGCCAGGAGGTG-3′ (forward) and 5′-GGTTCAGCAATAGCCGCAGA-3′ (reverse), and subcloned into pT7Blue (Promega), then integrated into the pCMV-Tag 2 vector (Stratagene) to synthesize FLAG-tagged TRADD. We transfected 293T cells with EGFP-Var DR3-D159G and/or His-Var DR3, and observed apoptosis by immunofluorescence microscope.

The 293T cells were also transfected with His-DR3 and/or Var DR3-D159G, and cell lysates were assessed for caspase 8 activity and DR3 protein simultaneously after 24 h. Lysates were reacted with Ac-IETD-AMC substrate (Peptide Institute, Edmonton), and caspase activity was determined by the fluorescence of released AMC in a CytoFluor 4000 (ABI) fluorescence spectrophotometer. Arbitrary fluorescence units (ΔAFU) were calculated as: (caspase activity of DR3 vector) − (caspase activity of empty vector).

Lymphocytes (1 × 10^6^/ml) were stimulated with TL1A (200 ng/ml) for 16 h and observed for surface annexin V (Trevigen, Gaithersburg, MD) expression by flow cytometry. Lysates from stimulated lymphocytes pretreated with cycloheximide were incubated with TL1A (200 ng/ml: R&D Systems) for 8–16 h and subjected to SDS-PAGE, followed by Western blotting with anti-caspase 8 antibody (Cell Signaling Technology, Danvers, MA). Human lymphocytes were treated with agonistic anti-DR3 (SS) antibody, anti-Fas monoclonal antibody (agonistic antibody CH-11; MBL, Nagoya, Japan), or anti-Fas polyclonal antibody (neutralize antibody HPA027444; Funakoshi, Tokyo, Japan) for 48 h, and the percent of cell death was calculated.

### DR3 transgenic mouse

C57BL/6 mice were obtained from SLC (Hamamatsu, Japan). Mutant-type human DR3 cDNA (consisted of exon 1 to 5 with a point mutation of g.1755A→G; Asp-159, an intron 5 segment including stop codon) were ligated into downstream of the mouse H-2 promotor. The construct was injected into fertilized eggs of C57BL/6 mice.

Splenocyte and TF-1 cells were lysed by hypotonic buffer (25 mm Tris, pH 8.0, 1% Nonidet P-40, 150 mm NaCl, 1.5 mm EGTA, 0.5% sodium deoxycholate, 1 mm PMSF, and 10 mm sodium orthovanadate). After the cytoplasmic protein was clarified by centrifugation at 15,000 rpm for 10 min, nucleus protein samples were obtained from the centrifuged pellet. The precipitation was lysed with buffer containing 10 mm HEPES, pH 7.9, 1.5 mm MgCl_2_, 10 mm KCl, 1 mm PMSF, and 10 mm sodium orthovanadate. Samples were probed with antibodies to Caspase 3, PARP (Cell Signaling Technology), IκBα, NFκB/p65 (BD Bioscience), and TL1A (Santa Cruz).

Myelocytes were obtained from mice femurs and cultured in RPMI 1640 with 10% FBS. After overnight culture, cells were incubated with 25 ng/ml of mouse GM-CSF (Wako) for 72 h in serum-free medium (COSMEDIUM; Cosmo Bio, Tokyo, Japan) and subjected to Western blots probed with anti-DR3 (SS). The culture supernatants of mice myelocytes were concentrated using Centriplus concentrators (Millipore, Bedford, MA), followed by immunoprecipitation with anti-TL1A Abs (R&D Systems) and Western blot with anti-DR3 Abs (B65; Neomarkers Inc., Fremont, CA). TF-1A cells were stimulated with TL1A (300 ng ml) and culture supernatants of myelocytes from TG or littermate control mice overnight. Western blot of TF-1A cells were probed with anti-total PARP, and anti-cleaved PARP (Cell Signaling Technology).

For induction of CIA, bovine type II collagen (Koken, Tokyo, Japan) was dissolved in 0.1 m acetic acid solution and emulsified in an equal volume of Freund' complete adjuvant (Difco, Detroit, MI). 7-Week-old DBA1/B6 mice, generated by backcross of C57BL/B6 mice toward DBA1/J mice until F6, were immunized by 200 μl of the emulsion. 21 days after primary immunization, an additional injection was performed as a booster immunization. The arthritis score was evaluated by each paw according to the following grading scale: 0 = normal joint, 1 = 1 or 2 swollen joints or slight swelling of front and hind paws, 2 = more than 3 swollen joints or significant swelling of front and hind paws, 3 = extreme swelling of the entire paw. An arthritis score (range 0–12) was assigned to each mouse by summing the scores of each paw. For histological studies, anti-MMP3 antibody (ab53015, Abcam) was used for immunohistochemistry and the ApoTag Peroxidase In Situ Apoptosis Detection Kit (S7100, Millipore) was for TUNNEL staining.

## Author contributions

A. H. performed and analyzed most of the experiments and wrote the manuscript. Y. K., K. M., and H. K. performed DNA sequencing, genotyping, and study of DR3 assembly. K. Y. and M. M. performed study of splicing regulatory proteins. K. T., T. S., and H. K. assisted with the study of apoptosis and analysis of results. K. T. performed study of DR3 transgenic mice. K. K. performed genotyping. T. K., H. Y., and K. S. collected human samples. S. S. conceived the idea for the project, supervised the experiments, and wrote the manuscript. All authors contributed to the final manuscript.

## Supplementary Material

Supporting Information

## Footnotes

2 The abbreviations used are:

DR3: death receptor 3
RA: rheumatoid arthritis
LARD: lymphocyte-associated receptor of death
TNFR: tumor necrosis factor receptor
TRADD: TNFR-associated death domain protein
TL1A: TNF-like ligand 1A
SNP: single nucleotide polymorphism
PBL: peripheral blood lymphocyte
PMA: phorbol myristate acetate
PHA: phytohemagglutinin
hnRNP: heterogeneous nuclear ribonucleoprotein
NONO: non-POU domain-containing octamer-binding protein
BHK: baby hamster kidney
TG: transgenic
PARP: poly(ADP-ribose) polymerase
ACPA: anti-citrullinated protein antibody
Var: variant type
Ab: antibody
EGFP: enhanced green fluorescent protein
TRITC: tetramethylrhodamine isothiocyanate.

## References

[1] Tan K. B., Harrop J., Reddy M., Young P., Terrett J., Emery J., Moore G., and Truneh A. (1997) Characterization of a novel TNF-like ligand and recently described TNF ligand and TNF receptor superfamily genes and their constitutive and inducible expression in hematopoietic and non-hematopoietic cells. Gene 204, 35–46 10.1016/S0378-1119(97)00509-X 9434163

[2] Kitson J., Raven T., Jiang Y. P., Goeddel D. V., Giles K. M., Pun K. T., Grinham C. J., Brown R., and Farrow S. N. (1996) A death-domain-containing receptor that mediates apoptosis. Nature 384, 372–375 10.1038/384372a0 8934525

[3] Chinnaiyan A. M., O'Rourke K., Yu G. L., Lyons R. H., Garg M., Duan D. R., Xing L., Gentz R., Ni J., and Dixit V. M. (1996) Signal transduction by DR3, a death domain-containing receptor related to TNFR-1 and CD95. Science 274, 990–992 10.1126/science.274.5289.990 8875942

[4] Marsters S. A., Sheridan J. P., Donahue C. J., Pitti R. M., Gray C. L., Goddard A. D., Bauer K. D., and Ashkenazi A. (1996) Apo-3, a new member of the tumor necrosis factor receptor family, contains a death domain and activates apoptosis and NF-κB. Curr. Biol. 6, 1669–1676 10.1016/S0960-9822(02)70791-4 8994832

[5] Screaton G. R., Xu X. N., Olsen A. L., Cowper A. E., Tan R., and McMichael A. J., and Bell J. I. (1997) LARD: a new lymphoid-specific death domain containing receptor regulated by alternative pre-mRNA splicing. Proc. Natl. Acad. Sci. U.S.A. 94, 4615–4619 10.1073/pnas.94.9.4615 9114039 PMC20772

[6] Ashkenazi A., and Dixit V. M. (1998) Death receptors: signaling and modulation. Science 281, 1305–1308 10.1126/science.281.5381.1305 9721089

[7] Pobezinskaya Y. L., Choksi S., Morgan M. J., Cao X., and Liu Z. G. (2011) The adaptor protein TRADD is essential for TNF-like ligand 1A/death receptor 3 signaling. J. Immunol. 186, 5212–5216 10.4049/jimmunol.1002374 21421854 PMC3080469

[8] Cassatella M. A., da Silva G. P, Tinazzi I., Facchetti F., Scapini P., Calzetti F., Tamassia N., Wei P., Nardelli B., Roschke V., Vecchi A., Mantovani A., Bambara L. M., Edwards S. W., and Carletto A. (2007) Soluble TNF-like cytokine (TL1A) production by immune complexes stimulated monocytes in rheumatoid arthritis. J. Immunol. 178, 7325–7333 10.4049/jimmunol.178.11.7325 17513783

[9] Prehn J. L., Thomas L. S., Landers C. J., Yu Q. T., Michelsen K. S., and Targan S. R. (2007) The T cell costimulator TL1A is induced by FcγR signaling in human monocyte and dendritic cells. J. Immunol. 178, 4033–4038 10.4049/jimmunol.178.7.4033 17371957

[10] Bamias G., Martin C. 3rd, Marini M., Hoang S., Mishina M., Ross W. G., Sachedina M. A., Friel C. M., Mize J., Bickston S. J., Pizarro T. T., Wei P., and Cominelli F. (2003) Expression, localization, and functional activity of TL1A, a novel Th1-polarizing cytokine in inflammatory bowel disease. J. Immunol. 171, 4868–4874 10.4049/jimmunol.171.9.4868 14568967

[11] Bossen C., Ingold K., Tardivel A., Bodmer J. L., Gaide O., Hertig S., Ambrose C., Tschopp J., and Schneider P. (2006) Interactions of tumor necrosis factor (TNF) and TNF receptor family members in the mouse and human. J. Biol. Chem. 281, 13964–13971 10.1074/jbc.M601553200 16547002

[12] Siakavellas S. I., Sfikakis P. P., and Bamias G. (2015) The TL1A/DR3/DcR3 pathway in autoimmune rheumatic diseases. Semin. Arthritis Rheum. 45, 1–8 10.1016/j.semarthrit.2015.02.007 25887448

[13] Zhang J., Wang X., Fahmi H., Wojcik S., Fikes J., Yu Y., Wu J., and Luo H. (2009) Role of TL1A in the pathogenesis of rheumatoid arthritis. J. Immunol. 183, 5350–5357 10.4049/jimmunol.0802645 19786547

[14] Meylan F., Davidson T. S., Kahle E., Kinder M., Acharya K., Jankovic D., Bundoc V., Hodges M., Shevach E. M., Keane-Myers A., Wang E. C., and Siegel R. M. (2008) The TNF-family receptor DR3 is essential for diverse T cell-mediated inflammatory diseases. Immunity 29, 79–89 10.1016/j.immuni.2008.04.021 18571443 PMC2760084

[15] Migone T. S., Zhang J., Luo X., Zhuang L., Chen C., Hu B., Hong J. S., Perry J. W., Chen S. F., Zhou J. X., Cho Y. H., Ullrich S., Kanakaraj P., Carrell J., Boyd E., et al (2002) TL1A is a TNF-like ligand and functions as a T cell for DR3 and TR6/DcR3 costimulater. Immunity 16, 479–492 10.1016/S1074-7613(02)00283-2 11911831

[16] Jin S., Chin J., Seeber S., Niewoehner J., Weiser B., Beaucamp N., Woods J., Murphy C., Fanning A., Shanahan F., Nally K., Kajekar R., Salas A., Planell N., et al (2013) TL1A/TNFSF15 directly induces proinflammatory cytokines, including TNFα from CD3+CD161+ T cells to exacerbate gut inflammation. Mucosal. Immunol. 6, 886–899 10.1038/mi.2012.124 23250276

[17] Bamias G., Mishina M., Nyce M., Ross W. G., Kollias G., Rivera-Nieves J., Pizarro T. T., and Cominelli F. (2006) Role of TL1A and its receptor DR3 in two models of chronic murine ileitis. Proc. Natl. Acad. Sci. U.S.A. 103, 8441–8446 10.1073/pnas.0510903103 16698931 PMC1482511

[18] Yamazaki K., McGovern D., Ragoussis J., Paolucci M., Butler H., Jewell D., Cardon L., Takazoe M., Tanaka T., Ichimori T., Saito S., Sekine A., Iida A., Takahashi A., Tsunoda T., Lathrop M., and Nakamura Y. (2005) Single nucleotide polymorphisms in TNFSF15 confer susceptibility to Crohn's disease. Hum. Mol. Genet. 14, 3499–3506 10.1093/hmg/ddi379 16221758

[19] Okada Y., Yamazaki K., Umeno J., Takahashi A., Kumasaka N., Ashikawa K., Aoi T., Takazoe M., Matsui T., Hirano A., Matsumoto T., Kamatani N., Nakamura Y., Yamamoto K., and Kubo M. (2011) HLA-Cw*1202-B*5201-DRB1*1502 haplotype increases risk for ulcerative colitis but reduces risk for Crohn's disease. Gastroenterology 141, 864–871.e1–5 10.1053/j.gastro.2011.05.048 21699788

[20] Yamazaki K., Umeno J., Takahashi A., Hirano A., Johnson T. A., Kumasaka N., Morizono T., Hosono N., Kawaguchi T., Takazoe M., Yamada T., Suzuki Y., Tanaka H., Motoya S., Hosokawa M., et al (2013) A genome-wide association study identifies 2 susceptibility loci for Crohn's disease in a Japanese population. Gastroenterology 144, 781–788 10.1053/j.gastro.2012.12.021 23266558

[21] Jostins L., Ripke S., Weersma R. K., Duerr R. H., McGovern D. P., Hui K. Y., Lee J. C., Schumm L. P., Sharma Y., Anderson C. A., Essers J., Mitrovic M., Ning K., Cleynen I., Theatre E., et al (2012) Host-microbe interactions have shaped the genetic architecture of inflammatory bowel disease. Nature 491, 119–124 10.1038/nature11582 23128233 PMC3491803

[22] Kakuta Y., Ueki N., Kinouchi Y., Negoro K., Endo K., Nomura E., Takagi S., Takahashi S., and Shimosegawa T. (2009) TNFSF15 transcripts from risk haplotype for Crohn's disease are overexpressed in stimulated T cells. Hum. Mol. Genet. 18, 1089–1098 10.1093/hmg/ddp005 19124533

[23] Sun X., Zhao J., Liu R., Jia R., Sun L., Li X., and Li Z. (2013) Elevated serum and synovial fluid TNF-like ligand (TL1A) is associated with autoantibody production in patients with rheumatoid arthritis. Scand. J. Rheumatol. 42, 97–101 10.3109/03009742.2012.727026 23311967

[24] Bamias G., Siakavellas S. I., Stamatelopoulos K., Christopoulos P., Papamichael C., and Sfikakis P. P. (2008) Circulating levels of TNF-like cytokine 1A (TL1A) and its decoy receptor (DcR3) in rheumatoid arthritis. Clin. Immunol. 129, 249–255 10.1016/j.clim.2008.07.014 18757243

[25] Xiu Z., Shen H., Tian Y., Xia L., and Lu J. (2015) Serum and synovial fluid levels of tumor necrosis factor-like ligand 1A and decoy receptor 3 in rheumatoid arthritis. Cytokine 72, 185–189 10.1016/j.cyto.2014.12.026 25647275

[26] Bamias G., Stamatelopoulos K., Zampeli E., Protogerou A., Sigala F., Papamichael C., Christopoulos P., Kitas G. D., and Sfikakis P. P. (2013) Circulating levels of TNF-like cytokine 1A correlate with the progression of atheromatous lesions in patients with rheumatoid arthritis. Clin. Immunol. 147, 144–150 10.1016/j.clim.2013.03.002 23598291

[27] Bamias G., Evangelou K., Vergou T., Tsimaratou K., Kaltsa G., Antoniou C., Kotsinas A., Kim S., Gorgoulis V., Stratigos A. J., and Sfikakis P. P. (2011) Upregulation and nuclear localization of TNF-like cytokine 1A TL1A) and its receptors DR3 in psoriatic skin lesions. Exp. Dermatol. 20, 725–731 10.1111/j.1600-0625.2011.01304.x 21672030

[28] Li L., Fu L., Lu Y., Wang W., Liu H., Li F., and Chen T. (2014) TNF-like ligand 1A is associated with the pathogenesis of psoriasis vulgaris and contributes to IL-17 production in PBMCs. Arch. Dermatol. Res. 306, 927–932 10.1007/s00403-014-1497-z 25200589

[29] Aiba Y., Harada K., Komori A., Ito M., Shimoda S., Nakamura H., Nagaoka S., Abiru S., Migita K., Ishibashi H., Nakanuma Y., Nishida N., Kawashima M., Tokunaga K., Yatsuhashi H., and Nakamura M. (2014) Systemic and local expression levels of TNF-like ligand 1A and its decoy receptor 3 are increased in primary biliary cirrhosis. Liver Int. 34, 679–688 10.1111/liv.12296 24016146

[30] Nakamura M., Nishida N., Kawashima M., Aiba Y., Tanaka A., Yasunami M., Nakamura H., Komori A., Nakamuta M., Zeniya M., Hashimoto E., Ohira H., Yamamoto K., Onji M., Kaneko S., Honda M., et al (2012) Genome-wide association study identifies TNFSF15 and POU2AF1 as susceptibility loci for primary biliary cirrhosis in the Japanese population. Am. J. Hum. Genet. 91, 721–728 10.1016/j.ajhg.2012.08.010 23000144 PMC3484650

[31] Konsta M., Bamias G., Tektonidou M. G., Christopoulos P., Iliopoulos A., and Sfikakis P. P. (2013) Increased levels of soluble TNF-like receptor 1A in ankylosing spondylitis. Rheumatology (Oxford) 52, 448–451 10.1093/rheumatology/kes316 23204549

[32] Zinovieva E., Bourgain C., Kadi A., Letourneur F., Izac B., Said-Nahal R., Lebrun N., Cagnard N., Vigier A., Jacques S., Miceli-Richard C., Garchon H. J., Heath S., Charon C., Bacq D., et al (2009) Comprehensive linkage and association analyses identify haplotype, near to the TNFSF15 gene, significantly associated with spondyloarthritis. PLoS Genet. 5, e10000528 10.1371/journal.pgen.1000528 19543369 PMC2689651

[33] Fang L., Adkins B., Deyev V., and Podack E. R. (2008) 9 Essential role of TNF receptor superfamily 25 (TNFRSF25) in the development of allergic lung inflammation. J. Exp. Med. 205, 1037–1048 10.1084/jem.20072528 18411341 PMC2373837

[34] Schreiber T. H., Wolf D., Tsai M. S., Chirinos J., Deyev V. V., Gonzalez L., Malek T. R., Levy R. B., and Podack E. R. (2010) Therapeutic Treg expansion in mice by TNFRSF25 prevents allergic lung inflammation. J. Clin. Invest. 120, 3629–3640 10.1172/JCI42933 20890040 PMC2947231

[35] Arnett F. C., Edworthy S. M., Bloch D. A., McShane D. J., Fries J. F., Cooper N. S., Healey L. A., Kaplan S. R., Liang M. H., and Luthra H. S. (1998) The American Rheumatism Association 1987 revised criteria for the classification of rheumatoid arthritis. Arthritis Rheum. 31, 315–324 3358796 10.1002/art.1780310302

[36] Smith C. W., and Valcárcel V. (2000) Alternative pre-mRNA splicing: the logic of combinatorial control. Trends Biochem. Sci. 25, 381–388 10.1016/S0968-0004(00)01604-2 10916158

[37] Weyand C. M., Fujii H., Shao L., and Goronzy J. J. (2009) Rejuvenating the immune system in rheumatoid arthritis. Nat. Rev. Rheumatol. 5, 583–588 10.1038/nrrheum.2009.180 19798035

[38] McInnes I. B., and Schett G. (2011) The pathogenesis of rheumatoid arthritis. N. Engl. J. Med. 365, 2205–2219 10.1056/NEJMra1004965 22150039

[39] Vaishnaw A. K., Orlinick J. R., Chu J. L., Krammer P. H., Chao M. V., and Elkon K. B. (1999) The molecular basis for apoptosis defects in patients with CD95 (Fas/Apo-1) mutations. J. Clin. Invest. 103, 355–363 10.1172/JCI5121 9927496 PMC407903

[40] Fisher G. H., Rosenberg F. J., Straus S. E., Dale J. K., Middleton L. A., Lin A. Y., Strober W., Lenardo M. J., and Puck J. M. (1995) Dominant interfering Fas gene mutations impair apoptosis in a human autoimmune lymphoproliferative syndrome. Cell 81, 935–946 10.1016/0092-8674(95)90013-6 7540117

[41] Singer G. G., and Abbas A. K. (1994) The Fas antigen is involved in peripheral but not thymic deletion of T lymphocytes in T cell receptor transgenic mice. Immunity 1, 365–371 10.1016/1074-7613(94)90067-1 7533645

[42] Watanabe-Fukunaga R., Brannan C. I., Copeland N. G., Jenkins N. A., and Nagata S. (1992) Lymphoproliferation disorder in mice explained by defects in Fas antigen that mediates apoptosis. Nature 356, 314–337 10.1038/356314a0 1372394

[43] Takahashi T., Tanaka M., Brannan C. I., Jenkins N. A., Copeland N. G., Suda T., and Nagata S. (1994) Generalized lymphoproliferative disease in mice, caused by a point mutation in the Fas ligand. Cell 76, 969–976 10.1016/0092-8674(94)90375-1 7511063

[44] Kaufmann S. H., Desnoyers S., Ottaviano Y., Davidson N. E., and Poirier G. G. (1993) Specific proteolytic cleavage of poly(ADP-ribose) polymerase: an early marker of chemotherapy-induced apoptosis. Cancer Res. 53, 3976–3985 8358726

[45] Wu J., Wilson J., He J., Xiang L., Schur P. H., and Mountz J. D. (1996) Fas ligand mutation in a patient with systemic lupus erythematosus and lymphoproliferative disease. J. Clin. Invest. 98, 1107–1113 10.1172/JCI118892 8787672 PMC507531

[46] Patton J. G., Porro E. B., Galceran J., Tempst P., and Nadal-Ginard B. (1993) Cloning and characterization of PSF, a novel pre-mRNA splicing factor. Genes Dev. 7, 393–406 10.1101/gad.7.3.393 8449401

[47] Shav-Tai Y., and Zipori D. (2002) PSF and p54^nrb^/NonO- multi-functional nuclear proteins. FEBS Lett. 531, 109–114 10.1016/S0014-5793(02)03447-6 12417296

[48] Peng R., Dye B. T., Pérez I., Bernard D. C., Thompson A. B., and Patton J. G. (2002) PSF and p54nrb bind a conserved stem in U5 snRNA. RNA 8, 1334–1347 10.1017/S1355838202022070 12403470 PMC1370341

[49] Motta-Mena L. B., Heyd F., and Lynch K. W. (2010) Context-dependent regulatory mechanism of the splicing factor hnRNP L. Mol. Cell 37, 223–234 10.1016/j.molcel.2009.12.027 20122404 PMC2818868

[50] Fukao T., Horikawa R., Naiki Y., Tanaka T., Takayanagi M., Yamaguchi S., and Kondo N. (2010) A novel mutation (c. 951C>T) in an exonic splicing enhancer results in exon 10 skipping in the human mitochondrial acetoacetyl-CoA thiolase gene. Mol. Genet. Metab. 100, 339–344 10.1016/j.ymgme.2010.03.012 20488739

[51] Lu Z. X., Peng J., and Su B. (2007) A human-specific mutation leads to the origin of a novel splice form of neuropsin (KLK8), a gene involved in learning and memory. Hum. Mutat. 28, 978–984 10.1002/humu.20547 17487847

[52] Genetta T., Morisaki H., Morisaki T., and Holmes E. W. (2001) A novel bipartite intronic splicing enhancer promotes the inclusion of a mini-exon in the AMP deaminase 1 gene. J. Biol. Chem. 276, 25589–25597 10.1074/jbc.M011637200 11331279

[53] Rossbach O., Hung L. H., Schreiner S., Grishina I., Heiner M., Hui J., and Bindereif A. (2009) Auto- and cross-regulation of the hnRNP L proteins by alternative splicing. Mol. Cell Biol. 29, 1442–1451 10.1128/MCB.01689-08 19124611 PMC2648227

[54] van Doorn R., Dijkman R., Vermeer M. H., Starink T. M., Willemze R., and Tensen C. P. (2002) A novel splice variant of the *Fas* gene in patients with cutaneous T-cell lymphoma. Cancer Res. 62, 5389–5392 12359741

[55] Boldin M. P., Varfolomeev E. E., Pancer Z., Mett I. L., Camonis J. H., and Wallach D. (1995) A novel protein that interacts with the death domain of Fas/APO1 contains a sequence motif related to the death domain. J. Biol. Chem. 270, 7795–7798 10.1074/jbc.270.14.7795 7536190

[56] Borysenko C. W., Furey W. F., and Blair H. C. (2005) Comparative modeling of TNFRSF25 (DR3) predicts receptor destabilization by a mutation linked to rheumatoid arthritis. Biochem. Biophys. Res. Commun. 328, 794–799 10.1016/j.bbrc.2005.01.017 15694416

[57] Liu J. H., Wei S., Lamy T., Li Y., Epling-Burnette P. K., Djeu J. Y., and Loughran T. P. Jr. (2002) Blockade of Fas-dependent apoptosis by soluble Fas in LGL leukemia. Blood 100, 1449–1453 12149230

[58] Otsuki T., Sakaguchi H., Tomokuni A., Aikoh T., Matsuki T., Isozaki Y., Hyodah F., Kawakami Y., Kita S., and Ueki A. (2000) Detection of alternatively spliced variant messages of *Fas* gene and mutational from silicosis patients. Immunol. Lett. 72, 137–143 10.1016/S0165-2478(00)00177-2 10841950

[59] Osawa K., Takami N., Shiozawa K., Hashiramoto A., and Shiozawa S. (2004) Death receptor 3 (*DR3*) gene duplication in a chromosome region 1p36.3: gene duplication is more prevalent in rheumatoid arthritis patients. Gene Immun. 5, 439–443 10.1038/sj.gene.6364097 15241467

[60] Takami N., Osawa K., Miura Y., Komai K., Taniguchi M., Shiraishi M., Sato K., Iguchi T., Shiozawa K., Hashiramoto A., and Shiozawa S. (2006) The promoter region of death receptor 3 (DR3) is specifically hypermethylated in rheumatoid synovial cells. Arthritis Rheum. 54, 779–787 10.1002/art.21637 16508942

[61] Meusch U., Klingner M., Mathar C., Malysheva O., Baerwald C., Rossol M., and Wagner U. (2015) Autocrine cytokine-mediated deficiency of TRAIL-induced monocyte apoptosis in rheumatoid arthritis. Arthritis Rheumatol. 67, 1760–1765 10.1002/art.39138 10.1002/art.39138 25833292

[62] Hayashi S., Miura Y., Nishiyama T., Mitani M., Tateishi K., Sakai Y., Hashiramoto A., Kurosaka M., Shiozawa S., and Doita M. (2007) Decoy receptor 3 expressed in rheumatoid synovial fibroblasts protects the cells against Fas-induced apoptosis. Arthritis Rheumatol. 56, 1067–1075 10.1002/art.22494 17393415

