# Supplementary material for: A variant of death-receptor 3 associated with rheumatoid arthritis interferes with apoptosis-induction of T cell
Source: J Biol Chem. 2017 Nov 27;293(6):1933–43. doi: 10.1074/jbc.M117.798884 (PMC5808757; doi:10.1074/jbc.M117.798884)
Supplement: Supporting Information [file supp_293_6_1933__index.html]

A variant of death-receptor 3 associated with rheumatoid arthritis interferes with apoptosis-induction of T cell. — A variant of death-receptor 3 associated with rheumatoid arthritis interferes with apoptosis-induction of T cell. — A variant of death-receptor 3 associated with rheumatoid arthritis interferes with apoptosis-induction of T cell — A variant of DR3 interferes with apoptosis-induction — Supporting Information 

# A variant of death-receptor 3 associated with rheumatoid arthritis interferes with apoptosis-induction of T cell

## Supporting Information

- Supplemental Figure S1 (.docx, 215 KB) - Supplemental Figure S1
