# Supplementary figures and images for: A variant of death-receptor 3 associated with rheumatoid arthritis interferes with apoptosis-induction of T cell
Source: J Biol Chem. 2017 Nov 27;293(6):1933–43. doi: 10.1074/jbc.M117.798884 (PMC5808757; doi:10.1074/jbc.M117.798884)

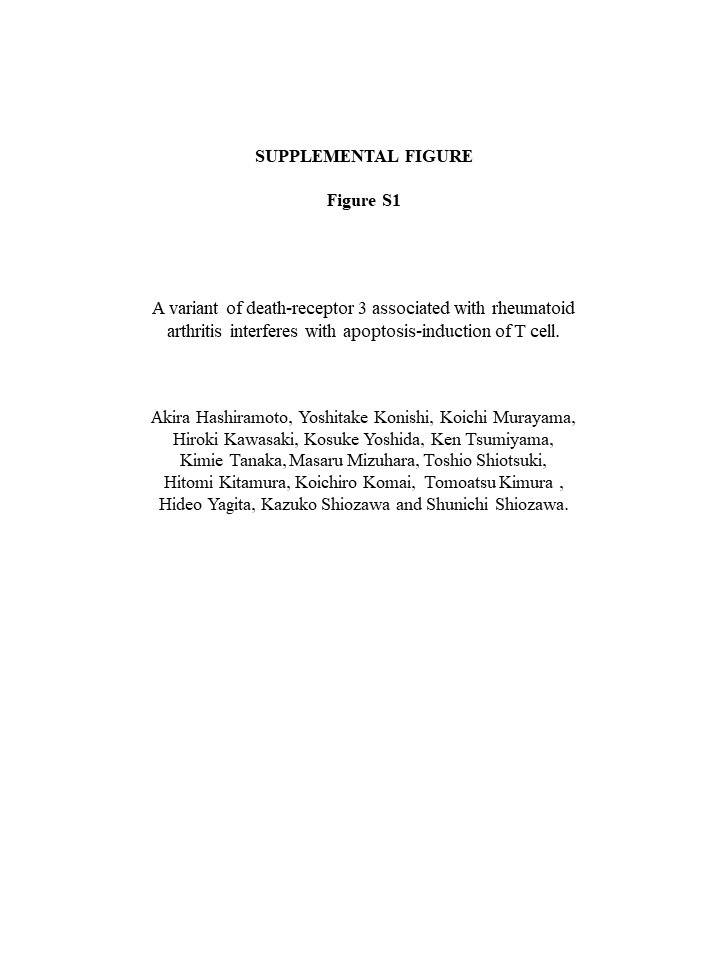


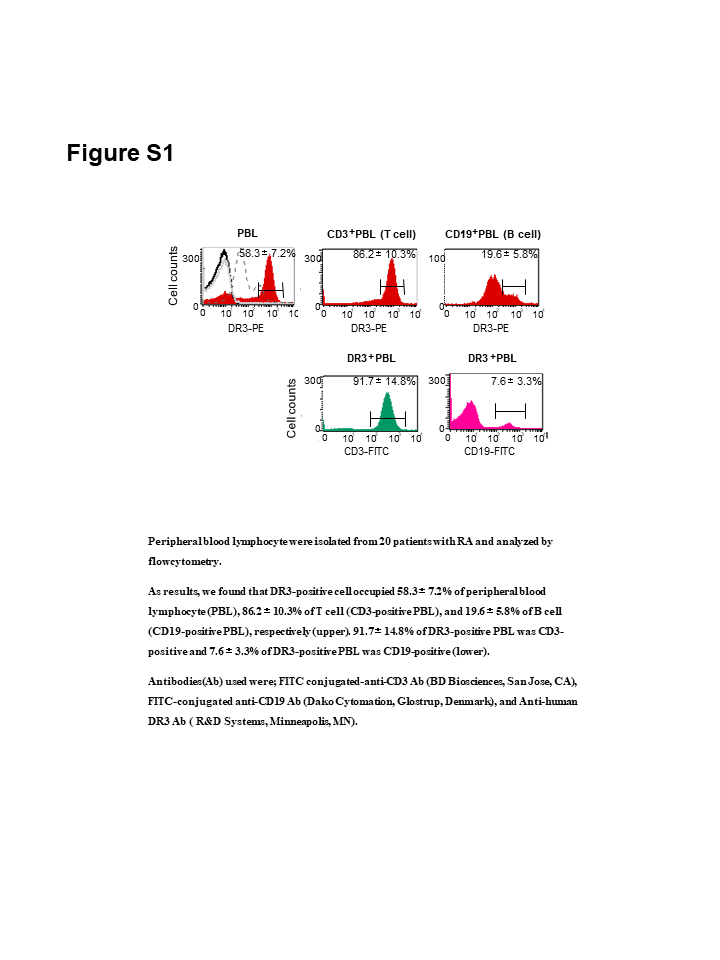

Supplement: Supporting Information [file 10.1074_M117.798884_jbc.M117.798884-1.docx]
